# Sustainable Corrosion Inhibition of Carbon Steel in Sulfuric Acid by Using *Neolentinus lepideus* Extract

**DOI:** 10.3390/molecules31213819

**Published:** 2026-10-22

**Authors:** Jose de Jesus Martinez-Gonzalez, Isaac Tello-Salgado, Roy Lopez-Sesenes, Jesus Porcayo-Calderon, America Maria Ramirez-Arteaga, Ana Karen Galvez-Larios, Jose Gonzalo Gonzalez-Rodriguez

**Affiliations:** 1 Department of Analytical Chemistry, Faculty of Chemical Sciences, Complutense University of Madrid, Plaza de las Ciencias 2, 28040 Madrid, Spain; josedmar@ucm.es; 2 Centro de Investigaciones Biologicas, Universidad Autonoma del Estado de Morelos, Av. Universidad 1001, Col. Chamilpa, Cuernavaca 62209, Mexico; isaac.tello@uaem.mx; 3 Facultad de Ciencias Quimicas e Ingenieria, Universidad Autonoma del Estado de Morelos, Av. Universidad 1001, Col. Chamilpa, Cuernavaca 62209, Mexico; rlopez@uaem.mx (R.L.-S.); america.rmz@uaem.mx (A.M.R.-A.); 4 Departamento de Ingenieria Quimica y Metalurgia, Universidad de Sonora, Hermosillo 83000, Mexico; 5 Centro de Investigación en Ingenieria y Ciencias Aplicadas (CIICAp), Universidad Autonoma del Estado de Morelos, Av. Universidad 1001, Col. Chamilpa, Cuernavaca 62209, Mexico; ana.galvez@uaem.mx

**Keywords:** carbon steel, green corrosion inhibitor, acid corrosion

## Abstract

In this work, the potential use of *Neolentinus lepideus* extract as a green corrosion inhibitor for carbon steel in acidic media was investigated, together with its prospects as a renewable and biologically derived corrosion-control material for potential industrial applications. To achieve this, an experimental investigation was conducted using gravimetric measurements, potentiodynamic polarization (PDP), and electrochemical impedance spectroscopy (EIS). The results demonstrated that the *N. lepideus* extract effectively suppressed the corrosion of 1018 carbon steel, with the inhibition efficiency increasing progressively with extract concentration and reaching a maximum value of 89% at 400 ppm. The potentiodynamic polarization curves revealed no evidence of passive film formation and indicated that the extract acted as a mixed-type corrosion inhibitor, with a more pronounced influence on the cathodic reaction. In the absence of the inhibitor, the corrosion process was predominantly governed by charge-transfer reactions and remained essentially under the same control mechanism upon the addition of 400 ppm of *Neolentinus lepideus* extract. However, at lower concentrations of 100 and 200 ppm, the corrosion process appeared to be affected by the adsorption and desorption of intermediate species at the steel/solution interface. The incorporation of *Neolentinus lepideus* extract into the corrosive medium resulted in an increase in the charge-transfer resistance and a concomitant decrease in the double-layer capacitance, supporting the formation of a protective interfacial film on the steel surface. The inhibitory action of the extract may be associated with its content of phenolic compounds, amides, and polysaccharides, whose heteroatoms and functional groups can interact with the steel surface and contribute to the formation of a protective adsorbed layer. The calculated Gibbs free energy values of adsorption (Δ*G*_ads_), which were less negative than −20 kJ mol^−1^, indicated that the adsorption of *Neolentinus lepideus* constituents onto the steel surface was predominantly physical in nature. Furthermore, the adsorption process was satisfactorily described by the Freundlich isotherm, suggesting heterogeneous adsorption involving surface sites with different adsorption energies.

## 1. Introduction

Steel is one of the most versatile engineering materials and is widely employed across numerous industrial sectors owing to its high mechanical strength, durability, and recyclability. Its applications include marine and offshore infrastructure, such as ship hulls, submarines, offshore platforms, oil rigs, and storage tanks [1]. Despite these advantageous properties, steel is highly susceptible to corrosion, which can result in significant economic losses, catastrophic failure and associated safety hazards [2,3,4,5] which occurs in industrial operations such as petroleum refining, acid pickling, chemical cleaning, and acid descaling, where mineral acids, including hydrochloric, phosphoric, nitric, and sulfuric acids, are extensively used.

Several approaches have been developed to mitigate metallic corrosion, but among these strategies, the use of corrosion inhibitors is particularly attractive because of its effectiveness and relatively low cost, especially for controlling corrosion in acidic media [6,7,8,9,10,11,12,13,14,15,16]. Conventional corrosion inhibitors employed in acidic environments are predominantly organic compounds, including amines, amides, imidazolines, and related molecules. Their inhibitive action is generally associated with adsorption onto the metal surface through interactions involving heteroatoms, such as nitrogen, sulfur, and oxygen, as well as aromatic rings and conjugated π-electron systems. However, these organic inhibitors have raised environmental and health concerns because certain compounds may exhibit toxicity, limited biodegradability, and adverse effects on living organisms and ecosystems.

Consequently, increasing research attention has been directed toward the development of low-cost, sustainable, and environmentally benign corrosion inhibitors derived from natural and renewable resources such as fruits, flowers, leaves, roots, fungi, and other biological sources, as well as alternative low-toxicity materials such as expired pharmaceutical compounds and carbon dots [17,18,19,20,21,22,23,24,25,26,27].

Some examples of these green corrosion inhibitors for steel in sulfuric acid include *Valeriana wallichii* root extract [17], waste feverfew root extract [18], aspirin [19], carbon dots [28], etc.

Collectively, these studies highlight the potential of natural products, pharmaceutical compounds, and carbon-based materials as alternatives to conventional corrosion inhibitors. Their inhibitive performance is closely related to the presence of functional groups and heteroatoms capable of interacting with metallic surfaces and promoting the formation of protective interfacial layers. Nevertheless, the development of additional inhibitor systems derived from abundant, renewable, and low-cost biological resources remains important for expanding the range of environmentally compatible corrosion-control technologies.

*Neolentinus lepideus* is a basidiomycete fungus belonging to the family Gloeophyllaceae. The species predominantly colonizes coniferous wood, particularly pine, and is commonly associated with decaying logs, processed timber, and preservative-treated wood, including railway ties. Its ability to produce lignocellulose-degrading enzymes enables it to colonize and degrade recalcitrant lignocellulosic substrates under relatively harsh environmental conditions, including dry and chemically treated wood. Consequently, *Neolentinus lepideus* has attracted interest in biotechnology because of its potential applications in biodegradation, biopulping, and bioremediation processes. Although the species may be consumed during its early developmental stages, its tough and fibrous texture limits its culinary use; nevertheless, it has traditionally been consumed in some regions of Mexico and Central America.

The availability of *Neolentinus lepideus* in Mexico represents an additional factor supporting the potential development of its extract as an environmentally friendly corrosion inhibitor. This edible mushroom occurs in Mexican coniferous forests, particularly in association with pine wood, and has a documented history of collection, consumption, and local commercialization in several regions of the country [29,30]. In Oaxaca, in the southern part of Mexico, for example, *Neolentinus lepideus* has been reported as an economically valuable wild mushroom and has also been subjected to controlled production. Furthermore, recent studies have demonstrated the feasibility of obtaining strains and cultivating this species on lignocellulosic residues. These characteristics suggest that *Neolentinus lepideus* could represent a renewable source of bioactive compounds for corrosion-inhibitor formulations.

The fungus has been reported to contain approximately 9.10% protein, 1.90% fat, and 8.90% dietary fiber, together with compounds such as vitamin C, ergosterol, α-tocopherol, γ-tocopherol, ascorbic acid, and phenolic compounds. These constituents have been associated with antioxidant, antibacterial, and potential anticancer activities [29,30]. The presence of phenolic compounds and other organic constituents containing heteroatoms and electron-donating functional groups is particularly relevant from the perspective of corrosion inhibition. Accordingly, the complex mixture of bioactive compounds present in *Neolentinus lepideus* may provide multiple adsorption sites capable of interacting with the metal/electrolyte interface and contributing to the formation of a protective film. As far as to the authors concern, there are no studies on the use of *Neolentinus lepideus* as corrosion inhibitor. Therefore, the present study was undertaken to investigate the potential use of *Neolentinus lepideus* extract as a green corrosion inhibitor for 1018 carbon steel in sulfuric acid.

## 2. Results and Discussion

### 2.1. Infrared Characterization

**Figure 1:**
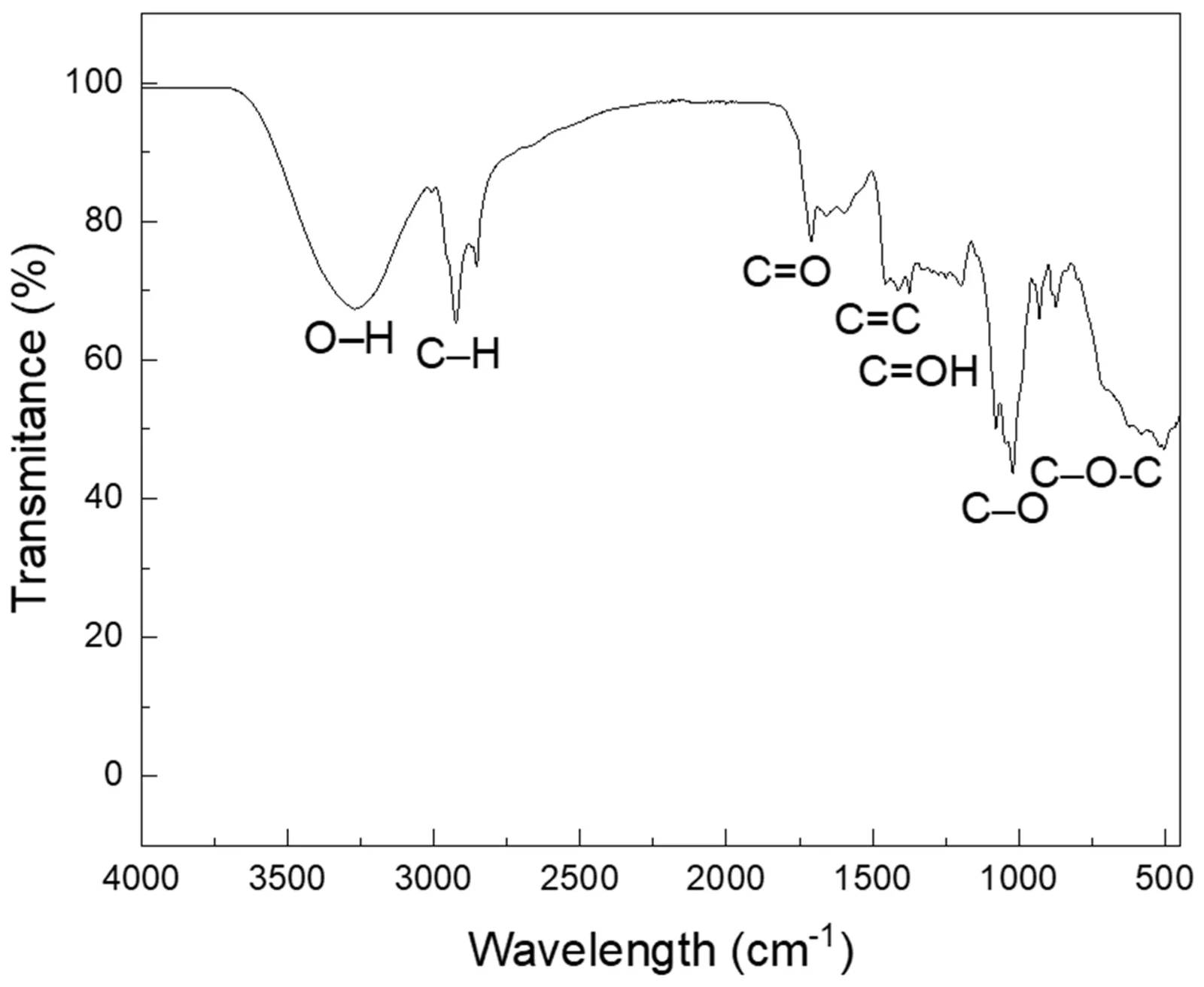
FTIR spectrum of *Neoentinus lepideus* extract.

The FTIR spectrum of *Neoentinus lepideus* is shown in Figure 1. The bands observed at 1453.6, 1412.1, and 1375.0 cm^−1^ can be mainly assigned to C–H deformation and bending vibrations of aliphatic CH_2_ and CH_3_ groups. The band near 1412 cm^−1^ may also include contributions from O–H bending and symmetric vibrations of carboxylate-containing species; therefore, overlapping contributions from different oxygen-containing functional groups cannot be excluded. Such assignments are consistent with FTIR spectra of polysaccharide-rich biological materials, in which CH_2_/CH_3_ deformation bands commonly occur near 1450 and 1370–1380 cm^−1^ [30].

Several absorption bands were observed in the 1248.7–1021.1 cm^−1^ region. This spectral interval is commonly referred to as the carbohydrate or polysaccharide region and contains overlapping vibrations associated with C–O and C–O–C stretching, C–C stretching, and C–OH bending. In particular, absorptions in the 1150–1050 cm^−1^ region are frequently associated with glycosidic C–O–C and C–O vibrations in polysaccharides [30,31]. Similar FTIR features have been reported for mushroom-derived polysaccharides, for which the 1200–950 cm^−1^ region is characteristic of carbohydrate structures [32,33]. Therefore, the group of bands observed in this region provides strong evidence for the presence of polysaccharide and other oxygenated carbohydrate constituents in the *N. lepideus* extract.

The absorption bands at 930.0 and 872.6 cm^−1^ may be associated with vibrations involving carbohydrate ring structures and glycosidic linkages. Bands in the vicinity of 900 cm^−1^ have been related to C–H deformation and vibrations involving glycosidic bonds, whereas absorptions near 870 cm^−1^ have also been reported for polysaccharide structures [32,33]. Although these bands cannot be considered diagnostic of a particular polysaccharide, their occurrence together with the intense absorption pattern in the 1200–950 cm^−1^ region further supports the presence of carbohydrate-derived constituents.

The relatively weak band at 658.0 cm^−1^ should be interpreted with greater caution. Low-wavenumber absorptions in this region may arise from several overlapping deformation, skeletal, or out-of-plane vibrational modes, and an unambiguous assignment to amide functionalities cannot be established from this band alone. Amide-containing compounds are more reliably identified by the characteristic amide I and amide II absorptions generally observed in the approximately 1600–1700 and 1500–1600 cm^−1^ regions, respectively [30,34]. Therefore, the band at 658.0 cm^−1^ is considered only a tentative indication of amide-related or other low-frequency vibrations. Nevertheless, when considered together with the O–H, C–H, C=O, and carbohydrate-region absorptions, the FTIR spectrum indicates that the *Neoentinus lepideus* extract contains a complex mixture of oxygenated organic constituents, including carbohydrate/polysaccharide components and potentially nitrogen-containing compounds.

Previous phytochemical investigations of *Neoentinus lepideus* have identified phenolic compounds such as protocatechuic, p-hydroxybenzoic, caffeic, vanillic, ferulic, salicylic, and trans-cinnamic acids, as well as scopoletin, together with α-tocopherol and ergosterol [33,34]. These compounds contain functional groups capable of interacting with the steel surface through oxygen- and, where present, nitrogen-containing donor atoms, while their aromatic and conjugated structures may also promote surface interactions through π-electron systems.

### 2.2. Gravimetric Tests

**Figure 2:**
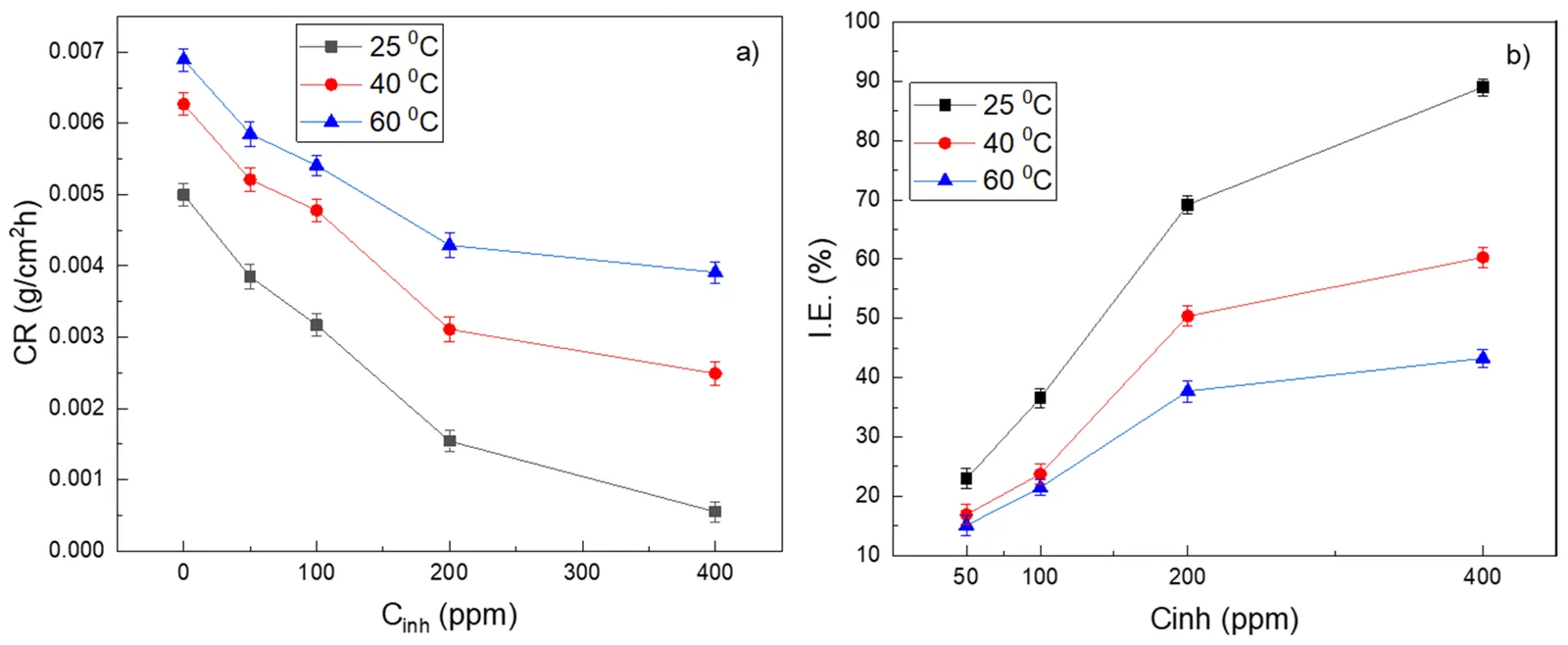
Effect of *Neolentinus lepideus* extract concentration on the (**a**) Corrosion rate and (**b**) Inhibitor efficiency for 1018 carbon steel in 0.5 M H_2_SO_4_.

The variation in corrosion rate (*CR*) and inhibition efficiency (IE) of 1018 carbon steel exposed to 0.5 M H_2_SO_4_ as a function of *Neolentinus lepideus* extract concentration at different temperatures is presented in Figure 2. As shown, the corrosion rate decreased progressively with increasing extract concentration, whereas an opposite trend was observed with increasing temperature. The maximum inhibition efficiency of 89% was achieved at an extract concentration of 400 ppm. The decrease in *CR* with increasing inhibitor concentration can be associated with the progressive adsorption of *Neolentinus lepideus* constituents onto the steel surface, resulting in increased surface coverage and the development of a protective interfacial film. This adsorbed layer can act as a barrier that limits the contact between the steel surface and the aggressive species present in the acidic electrolyte, thereby reducing the rate of the electrochemical reactions responsible for corrosion.

The increase in corrosion rate with temperature, accompanied by a decrease in inhibition efficiency, suggests that the protective effect of the extract becomes less pronounced at elevated temperatures. This behavior may be related to enhanced molecular motion and increased desorption of physically adsorbed inhibitor species from the steel surface, which can reduce the surface coverage of the protective layer. The temperature dependence of the inhibition efficiency therefore provides additional evidence regarding the nature and stability of the interaction between the extract constituents and the steel/electrolyte interface.

The inhibition efficiency obtained in the present study is comparable with values reported for other environmentally friendly corrosion inhibitors investigated in acidic media. Haldhar et al. [17] evaluated *Valeriana wallichii* root extract as a corrosion inhibitor for mild steel in 0.5 M H_2_SO_4_ and reported a maximum inhibition efficiency of 93.4% at an extract concentration of 500 ppm. Similarly, Zhou et al. [18] investigated waste feverfew root extract under comparable acidic conditions and reported a maximum inhibition efficiency of 96% at 400 ppm. Ikeuba et al. [19] examined expired aspirin as a corrosion inhibitor for carbon steel in 0.5 M H_2_SO_4_ and obtained a maximum inhibition efficiency of 79% at 800 ppm. In the present study, *Neolentinus lepideus* extract provided an inhibition efficiency of 89% at 400 ppm, which falls within the range reported for several bio-based and other environmentally friendly corrosion inhibitors for steel in acidic environments. Increasing the inhibitor concentration to 500 ppm might have resulted in a modest increase in inhibition efficiency, estimated at approximately 5–6 percentage points relative to that obtained at 400 ppm. However, such an improvement would be considered insufficient to justify the additional inhibitor concentration. Therefore, 400 ppm was selected as the optimum concentration for the present study. These results, as summarized in Table 1, indicate that *Neolentinus lepideus* extract can provide substantial corrosion protection at a relatively low concentration, highlighting its potential as a bio-derived inhibitor for carbon steel in sulfuric acid solutions.

### 2.3. Thermodynamic Parameters

**Figure 3:**
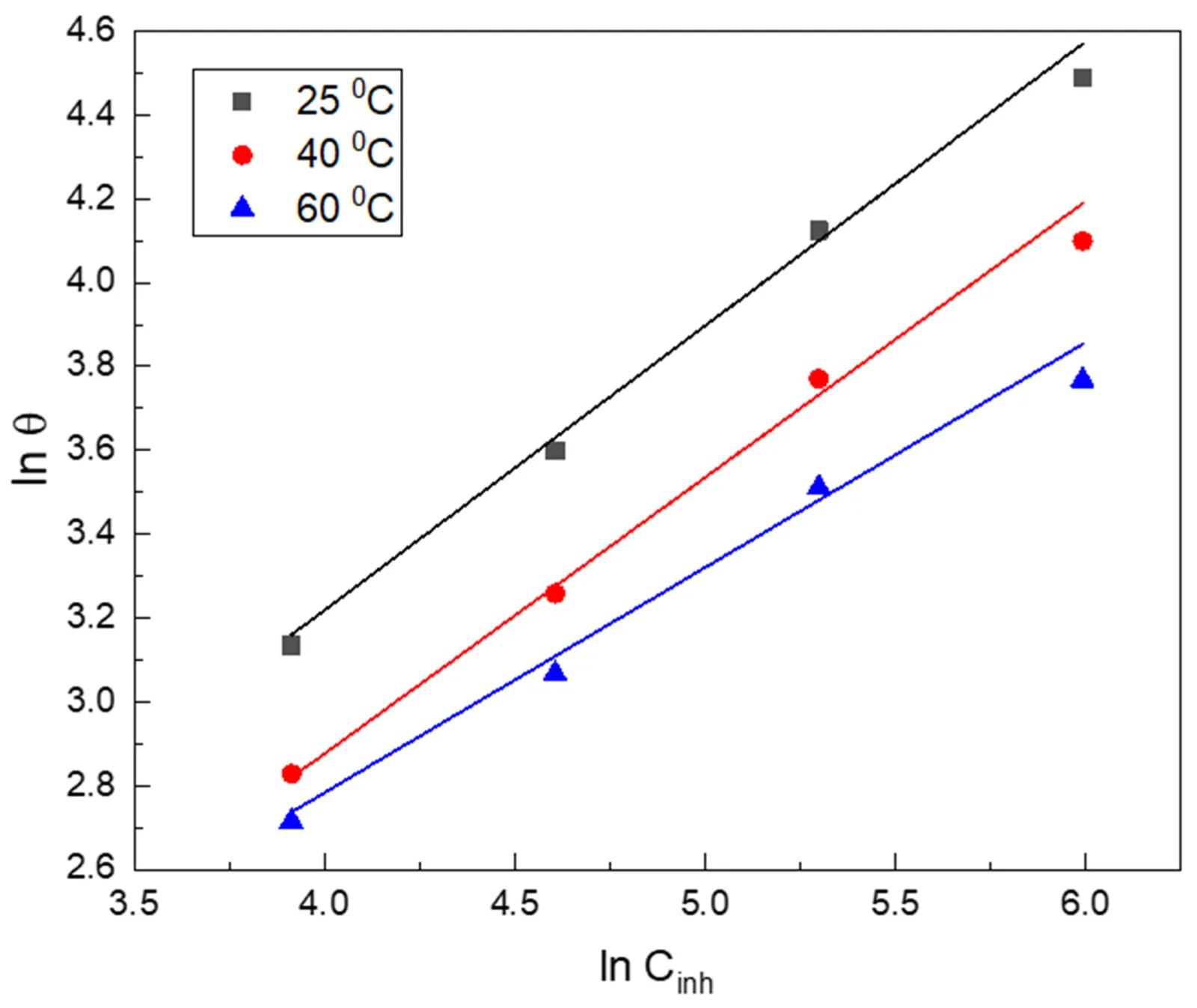
Freundich adsorption isotherm for 1018 carbon steel in 0.5 M H_2_SO_4_ in presence of *Neolentinus lepideus* extract.

As discussed above, the corrosion-inhibition performance of *Neolentinus lepideus* extract toward carbon steel in sulfuric acid can be primarily associated with the adsorption of its constituent molecules onto the steel surface, resulting in the formation of a protective interfacial film. To further elucidate the adsorption behavior of the extract and gain insight into the interactions between its constituent species and the steel surface, several adsorption isotherm models were examined. Among the models evaluated, the Freundlich isotherm provided the best correlation with the experimental data, as indicated by the highest correlation coefficient (R^2^) obtained, as shown in Figure 3. This result suggests that the adsorption of *Neolentinus lepideus* constituents occurs on a heterogeneous steel surface involving adsorption sites with different energies. The mathematical expression of the Freundlich adsorption isotherm can be written as follows:

ln θ = ln K_ads_ + nln C_inh_ (1)

where K_ads_ is the adsorption equilibrium constant, C_inh_ is the inhibitor concentration, and n is a dimensionless constant related to the characteristics and heterogeneity of the adsorbed molecules, with values typically ranging from zero to unity. The Langmuir model assumes, in its simplest form, a relatively homogeneous surface with equivalent adsorption sites, one adsorbed species per site, and no significant lateral interactions between adsorbed molecules [35]. The Freundlich model is empirical and is better suited to energetically heterogeneous surfaces, in which adsorption sites can have different affinities for the adsorbate. This is especially relevant to corrosion because a real steel surface is not perfectly homogeneous. It may contain different crystallographic orientations, grain boundaries, inclusions, defects and dislocations, etc. For a natural extract such as *Neolentinus lepideus*, this heterogeneity becomes even more important because the extract contains multiple classes of compounds—e.g., phenolic compounds, amides, polysaccharide-related species and other oxygen- and nitrogen-containing constituents—whose adsorption affinities toward the steel/electrolyte interface can differ [36]. In Equation (3) n is the **slope** of the linear plot and is an empirical parameter describing the concentration dependence of adsorption.

A plot of ln θ versus ln C_inh_ allows the determination of the Freundlich parameters, including K_ads_. Subsequently, the standard Gibbs free energy of adsorption, $∆G_{\mathrm{ads}}^{0}$, can be estimated from the relationship between K_ads_ and $∆G_{\mathrm{ads}}^{0}$, providing information about the spontaneity and nature of the adsorption process,

(2) $${\Delta G}_{\mathrm{ads}}^{0}=-\mathrm{RT}\;\mathrm{In}(10^{6}K_{\mathrm{ads}})$$

where *T* is the absolute temperature, *R* is the universal gas constant, and 10^6^ represents the molar concentration of water in the solution and is mainly a unit-conversion/standard-state correction, not a physical factor associated with adsorption itself, and is introduced in the conventional corrosion-literature treatment to transform the concentration-based K_ads_ into the numerical form used for the standard-state equilibrium constant.

For *Neolentinus lepideus* extract, K_ads_ should not be interpreted as a conventional thermodynamic equilibrium constant for a single molecular species, because the extract contains multiple compounds with different molecular masses and adsorption affinities. K_ads_, whose units are L mg^−1^ represents an empirical or apparent adsorption constant that characterizes the affinity of the overall extract for the steel surface under the experimental conditions.

The calculated values of Δ*G*_ads_ were −19.3, −16.7, and −14.2 kJ mol^−1^ at 25, 40, and 60 °C, respectively. In general, Δ*G*_ads_ values less negative than approximately −20 kJ mol^−1^ are commonly associated with predominantly physical adsorption, whereas values more negative than approximately −40 kJ mol^−1^ are generally considered indicative of chemisorption involving stronger interactions between inhibitor molecules and the metal surface. Accordingly, the Δ*G*_ads_ values obtained in the present study suggest that the adsorption of *Neolentinus lepideus* constituents onto the carbon steel surface is predominantly physical in nature. Because the *Neolentinus lepideus* extract is a complex mixture of organic constituents with no single well-defined molecular weight, the adsorption constant obtained from the Freundlich model should be regarded as an apparent or effective adsorption constant representing the collective adsorption behavior of the extract constituents. Accordingly, the corresponding Δ*G*_ads_ values should be interpreted as apparent Gibbs free energies of adsorption rather than rigorous molecular-level thermodynamic quantities.

This interpretation indicates that the inhibitor species are likely retained at the metal/electrolyte interface primarily through relatively weak interactions, including electrostatic forces and van der Waals interactions, rather than through the formation of strong chemical bonds with the steel surface. The progressive increase in Δ*G*_ads_ toward less negative values with increasing temperature may also indicate a decrease in the stability of the adsorbed layer at elevated temperatures, consistent with the experimentally observed decrease in inhibition efficiency.

**Figure 4:**
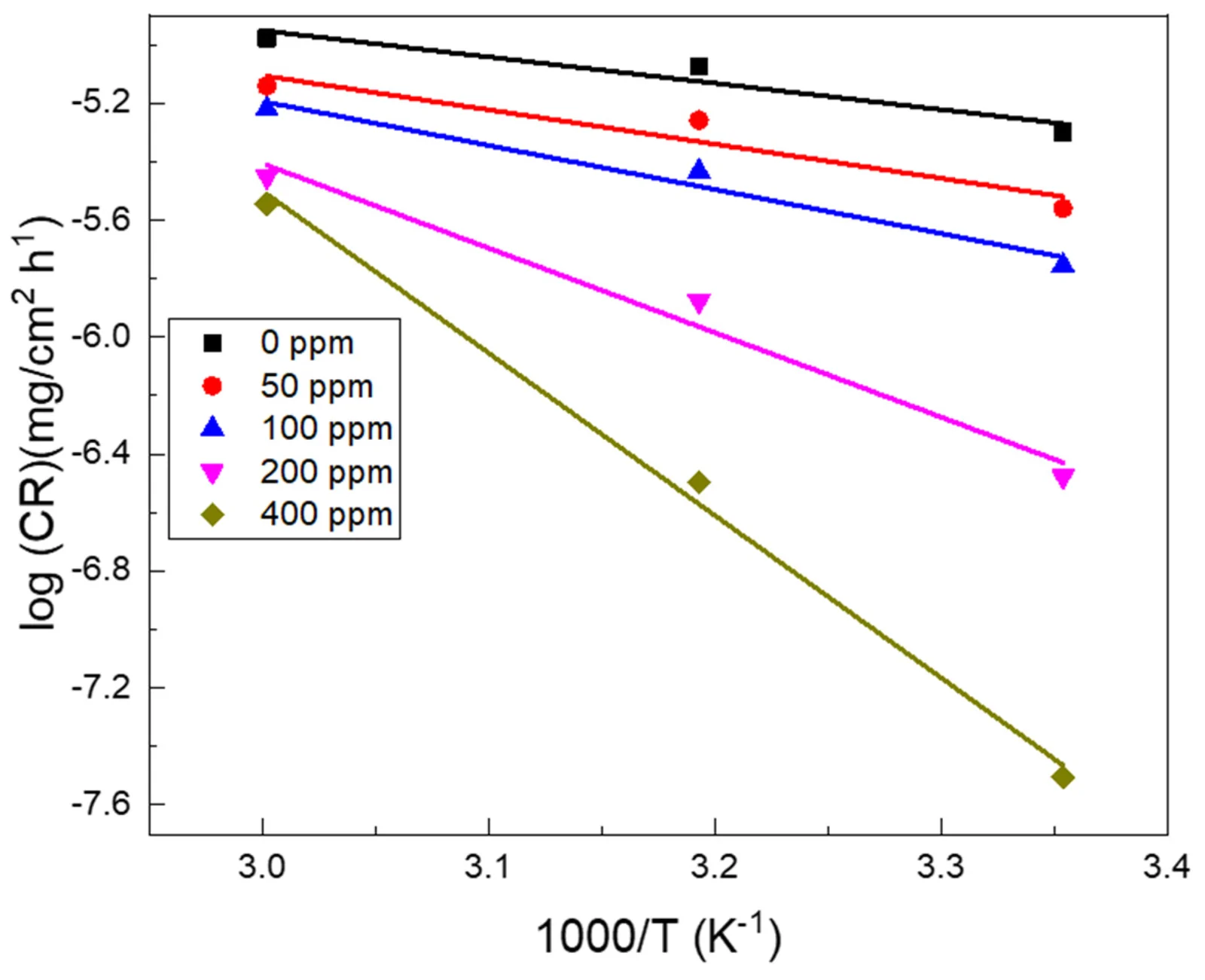
Log (*CR*) vs. 1000/*T* for 1018 carbon steel in 0.5 M H_2_SO_4_ in the presence of *Neolentinus lepideus* extract at different concentrations.

**Figure 5:**
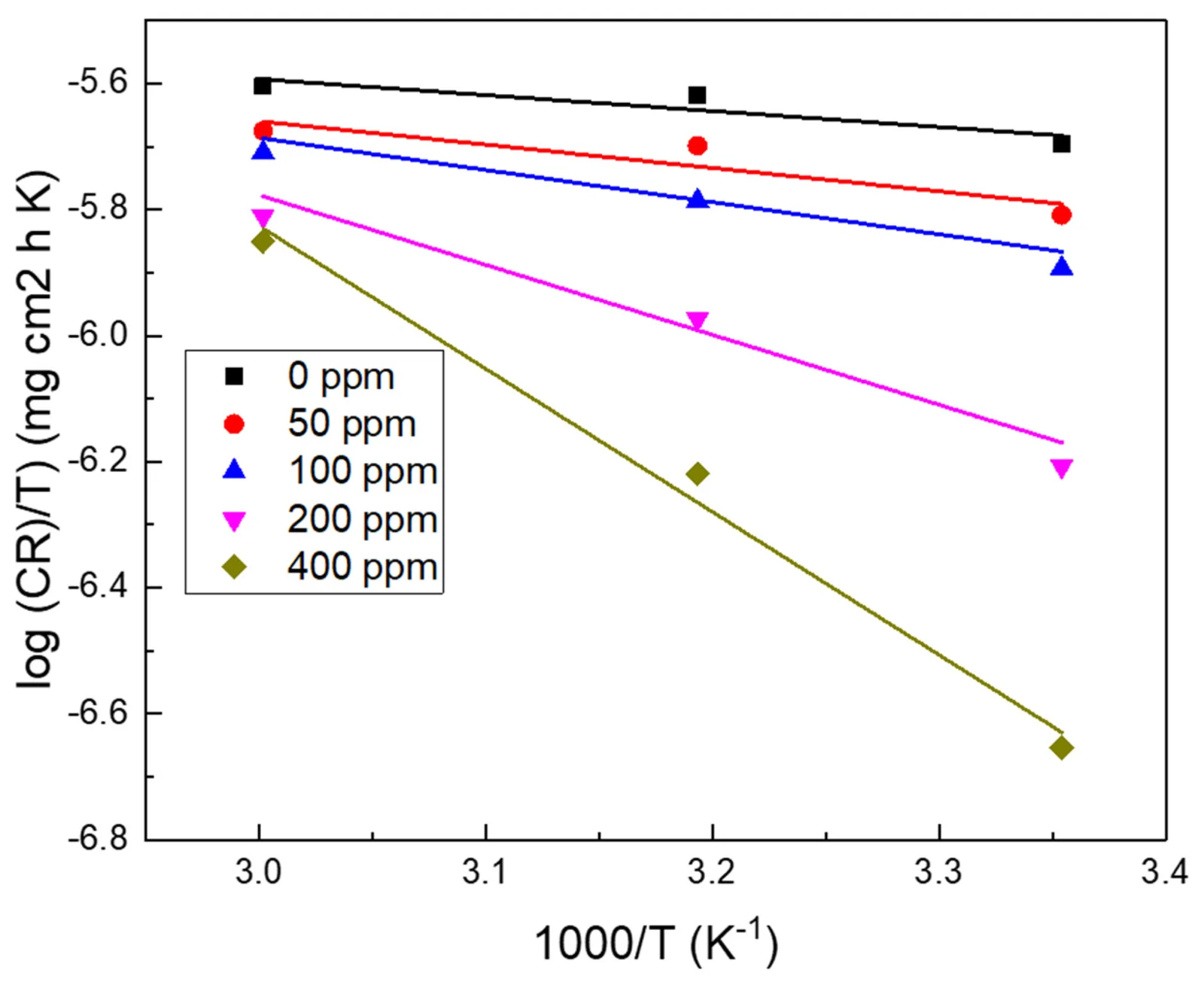
Log (*CR*/*T*) vs. 1000/*T* for 1018 carbon steel in 0.5 M H_2_SO_4_ in the presence of *Neolentinus lepideus* extract at different concentrations.

To further elucidate the effect of *Neolentinus lepideus* extract on the corrosion process, the apparent activation energy (*E_a_*) was determined for both the uninhibited and inhibited systems. For this purpose, the Arrhenius equation, which relates the corrosion rate (*CR*) to the absolute temperature (*T*), was employed as follows:

(3) $$\mathrm{CR}=Ae^{-\frac{E_{a}}{\mathrm{RT}}}$$

where *R* stands for the universal gas constant and *A* is a pre-exponential factor. A plot of log *CR* versus 1000/*T*, Figure 4, should be a straight line where the *E_a_* value could be obtained from its slope. On the other side, enthalpy and entropy values, ΔH* and ΔS* respectively, can be obtained by using the Eyring transition state equation:

(4) $$\mathrm{CR}=\frac{\mathrm{RT}}{\mathrm{Nh}}e^{\frac{∆S^{*}}{\mathrm{RT}}}e^{-\frac{∆H^{*}}{\mathrm{RT}}}$$

where *N* is the Avogadro’s number and *h* is the Planck-Boltzmann constant. From the slope we can calculate ΔH* whereas ΔS* can be obtained from the intercept of the log (*CR*/*T*) versus (1000/*T*) plots from Figure 5.

Table 2 summarizes the calculated values of the activation energy (*E_a_*), enthalpy of activation (ΔH*), and entropy of activation (ΔS*) for 1018 carbon steel in 0.5 M H_2_SO_4_ in the absence and presence of *Neolentinus lepideus* extract. As shown, *E_a_* increased progressively with increasing extract concentration. This increase indicates that the presence of the inhibitor raises the apparent energy barrier associated with the corrosion process, thereby hindering the electrochemical dissolution of the steel. Such behavior is consistent with the adsorption of *Neolentinus lepideus* constituents at the steel/electrolyte interface, which can reduce the availability of active sites for the electrochemical reactions involved in corrosion.

Similarly, ΔH* increased with increasing extract concentration, while remaining positive for all investigated systems. The positive ΔH* values indicate that the corrosion process is endothermic under the experimental conditions, meaning that the activated corrosion process requires energy input. The increase in ΔH* in the presence of the inhibitor suggests that the energy demand associated with the formation of the activated state becomes greater as the concentration of adsorbed inhibitor species increases. This behavior is consistent with the development of an interfacial barrier that makes the progression of the corrosion reaction less favorable.

The entropy of activation, ΔS* also increased with increasing *Neolentinus lepideus* extract concentration. The positive change in ΔS* may be related, at least in part, to modifications of the interfacial organization resulting from the adsorption of organic constituents of the extract. In the uninhibited solution, water molecules at the steel/electrolyte interface can exhibit a relatively ordered arrangement as a consequence of their interaction with the metallic surface. As inhibitor concentration increases, the adsorption of organic species may progressively displace some of these interfacial water molecules. Their subsequent release into the bulk solution provides greater translational and configurational freedom, which can contribute to an increase in the entropy associated with the activated corrosion process. Thus, the observed variation in ΔS* suggests that adsorption of *Neolentinus lepideus* constituents is accompanied by significant changes in the molecular organization of the steel/electrolyte interface.

### 2.4. Electrochemical Measurements

#### 2.4.1. Open Circuit Potential

**Figure 6:**
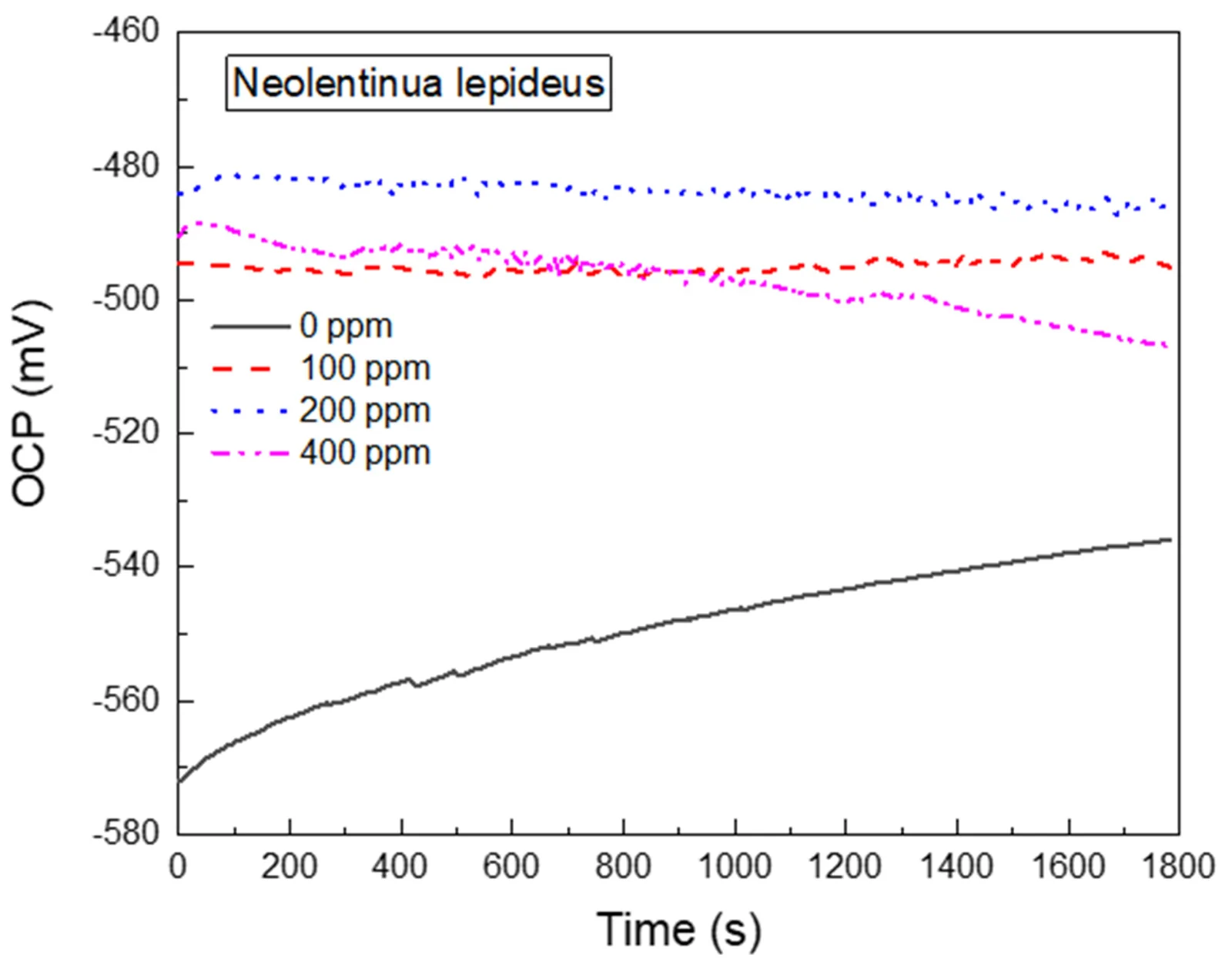
Variation in the OCP value with time for 1018 carbon steel in 0.5 M H_2_SO_4_ in the presence of *Neolentinus lepideus* extract at different concentrations.

The variation in the open-circuit potential (OCP) of AISI 1018 carbon steel immersed in 0.5 M H_2_SO_4_ containing different concentrations of *Neolentinus lepideus* extract is presented in Figure 6. In the uninhibited solution, the steel exhibited the most negative OCP, approximately −570 mV, indicating a relatively active electrochemical state at the beginning of immersion. This behavior can be associated with the direct exposure of the steel surface to the aggressive acidic electrolyte and the absence of a well-developed protective interfacial layer during the initial stage of exposure. As the immersion time increased, the OCP gradually shifted toward more positive values, reaching approximately −540 mV. This positive displacement suggests a progressive modification of the steel/solution interface, which may be associated with the accumulation of corrosion products and/or the development of a partially protective surface layer during exposure. Under sulfuric acid conditions, iron-containing corrosion products may include ferrous sulfate and hydrated iron sulfate species, although their specific nature cannot be established solely from the OCP measurements. The observed positive shift therefore indicates a gradual change in the interfacial electrochemical conditions as the corrosion process proceeds.

Upon addition of *Neolentinus lepideus* extract, the OCP shifted toward more positive (nobler) potentials and remained relatively stable throughout the monitoring period. This behavior indicates a modification of the steel/solution interface, which may be associated with the adsorption of inhibitor constituents and the subsequent development of a protective interfacial layer. The relatively stable OCP values in the presence of the extract suggest that the adsorbed species provide a persistent barrier that modifies the electrochemical activity of the AISI 1018 carbon steel surface and contributes to its enhanced corrosion resistance in the sulfuric acid medium. The maximum displacement of the OCP relative to the uninhibited system was less than 85 mV. According to the commonly used criterion based on the magnitude of the OCP displacement, such a relatively small shift does not correspond to the characteristic potential displacement generally associated with a predominantly anodic or cathodic inhibitor. Therefore, *Neolentinus lepideus* extract can be considered a mixed-type corrosion inhibitor, influencing both the anodic metal-dissolution reaction and the cathodic reaction(s) [22,23,24,25]. Nevertheless, the classification of the inhibitor type is more reliably established from the potentiodynamic polarization data, whereas OCP measurements primarily provide information on changes in the electrochemical state of the steel/solution interface.

#### 2.4.2. Potentiodynamic Polarization Curves

**Figure 7:**
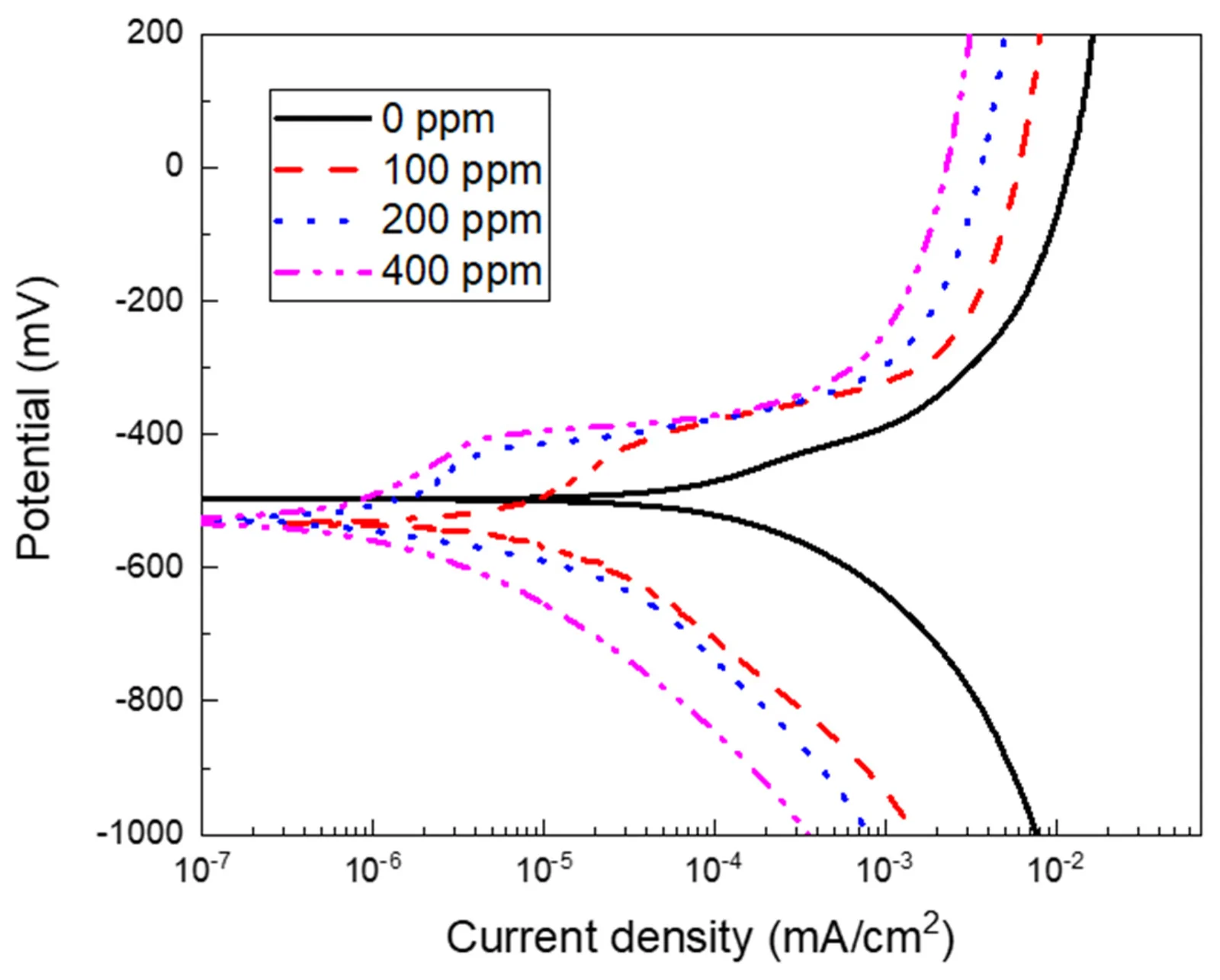
Effect of *Neolentinus lepideus* extract concentration on the polarization curves for 1018 carbon steel in 0.5 M H_2_SO_4_.

In the above section, some thermodynamic parameters for the corrosion of 1018 carbon steel in sulfuric acid in the absence and presence of *Neolentinus lepideus* were obtained, in order to have some kinetic parameters, the effect of *Neolentinus lepideus* concentration on the polarization curves for carbon steel are shown on Figure 7 whereas kinetic parameters such as E_corr_, I_corr_, polarization resistance, R_p_, anodic and cathodic Tafel slopes, β_a_ and β_c_ respectively, as well as the inhibitor efficiency and steel surface area covered by the inhibitor, θ are given on Table 3. The value for θ was obtained by dividing the inhibitor efficiency value by 100, whereas R_p_ was calculated by using the Stern-Geary equation:

R_p_ = β_a_ · β_c_/(2.23β_a_ + β_c_) · 1/I_corr_ (5)

As shown by the polarization curves presented in Figure 7, no distinct passive region was observed on the anodic branch, indicating that AISI 1018 carbon steel exhibited predominantly active dissolution behavior in the investigated 0.5 M H_2_SO_4_ medium. This behavior may be associated with the formation and/or accumulation of iron-containing corrosion products at the steel/solution interface. Depending on the local electrochemical conditions and exposure time, reported corrosion products in sulfuric acid media may include FeSO_4_ and hydrated ferrous sulfate species (e.g., FeSO_4_·7H_2_O, melanterite), as well as iron hydroxides and iron oxides/oxyhydroxides such as Fe(OH)_2_, Fe_3_O_4_ (magnetite), Fe_2_O_3_, and FeOOH [17,18,19,20,21,22,23,24,25]. However, the specific nature and relative abundance of these species cannot be established solely from the polarization measurements and would require complementary surface-characterization techniques.

Upon addition of *Neolentinus lepideus* extract, the E_corr_ value shifted toward more negative values, from approximately −490 mV in the uninhibited solution to approximately −530 mV in the presence of the extract. This displacement indicates a modification of the electrochemical behavior of the steel surface upon exposure to the inhibitor. Nevertheless, the magnitude and direction of the E_corr_ shift alone are not sufficient to determine the inhibitor type; therefore, the relative effects of the extract on the anodic and cathodic reactions should be assessed from the changes in the corresponding polarization branches and electrochemical parameters.

The principal anodic reaction is:

Fe → Fe^2+^ + 2e^−^ (6)

while hydrogen evolution is generally the dominant cathodic reaction:

2H^+^ +2e^−^ → H_2_ (7)

Oxygen reduction can also occur on AISI 1018 carbon steel exposed to 0.5 M H_2_SO_4_ at 25 °C, provided the solution contains dissolved oxygen:

O_2_ + 4H^+^ + 4e^−^ → 2H_2_O (8)

However, in this strongly acidic medium, the cathodic reaction is expected to be predominantly associated with hydrogen evolution, although oxygen reduction may also contribute under naturally aerated conditions. From Table 3 we can see that an increase in the *Neolentinus lepideus* extract concentration brings an increase in the polarization resistance value, R_p_, which, according to Equation (7) should result in a decrease in the corrosion current density value, I_corr_. The progressive increase in polarization resistance with increasing *Neolentinus lepideus* extract concentration can be attributed to the enhanced adsorption of the extract constituents onto the carbon-steel surface. As the inhibitor concentration increases, a greater number of active organic molecules become available to occupy electrochemically active sites, promoting the formation of a more continuous and compact adsorbed protective film. This interfacial layer acts as a barrier to the transport of corrosive species and reduces the effective area available for both anodic iron dissolution and cathodic reduction reactions. Consequently, the kinetics of the electrochemical corrosion processes are progressively suppressed, leading to an increase in R_p_. The higher polarization resistance therefore indicates a lower corrosion rate and supports the enhanced inhibition efficiency observed at increasing extract concentrations.

As shown in Figure 7, the addition of *Neolentinus lepideus* extract resulted in a pronounced decrease in both anodic and cathodic current densities, accompanied by a substantial reduction in the corrosion current density (I_corr_). The lowest I_corr_ value was recorded at an extract concentration of 400 ppm, corresponding to a reduction of approximately two orders of magnitude compared with the uninhibited solution. This behavior indicates that the extract effectively suppressed the electrochemical corrosion reactions at the steel/solution interface. The inhibition effect can be primarily associated with the adsorption of organic constituents of the extract onto the steel surface, resulting in the formation of a protective interfacial layer that limits the interaction between the metal surface and the aggressive acidic electrolyte.

Some differences can be observed between the inhibitor efficiency values reported by the weight loss tests, 89%, and the potentiodynamic polarization plots, 99%. Weight-loss measurements typically represent the average corrosion rate over a relatively long period (e.g., 72 h), whereas potentiodynamic polarization plots provides an estimate of the corrosion rate from the electrochemical behavior measured over a few minutes. Thus, weight loss reflects the overall long-term behavior of the inhibitor, including adsorption, desorption, degradation, and possible changes in the surface film. potentiodynamic polarization plots essentially provides a near-instantaneous estimate of the corrosion rate around the corrosion potential. For natural extracts such as *Neolentinus lepideus*, the situation can be particularly important because the inhibitor contains multiple organic compounds. During a long immersion period, some constituents can adsorb strongly, while others can desorb, dissolve, react with the metal surface, or undergo chemical transformation. Inhibitor adsorption is not necessarily instantaneous. Immediately after immersion, the surface may not be completely covered. During a 72 h weight-loss experiment, the inhibitor has considerably more time to establish an adsorbed protective layer. Calculated values for I_corr_ depends on the quality of the Tafel regions and on the validity of the extrapolation. If the polarization curves do not exhibit well-defined linear Tafel regions, the calculated I_corr_ can have significant uncertainty. This is especially relevant for green inhibitors, because adsorption films can produce deviations from ideal Tafel behavior.

As summarized in Table 3, the addition of *Neolentinus lepideus* extract affected both the anodic and cathodic Tafel slopes, although the variation in the cathodic slope was more pronounced. This behavior indicates that the extract influences both partial electrochemical reactions and can therefore be classified as a mixed-type corrosion inhibitor, in agreement with the OCP results, with a comparatively stronger effect on the cathodic process. Under the experimental conditions employed, hydrogen evolution is expected to constitute the principal cathodic reaction in the acidic electrolyte, whereas oxygen reduction may provide an additional cathodic contribution in the presence of dissolved oxygen.

The inhibition of the anodic reaction can be attributed to the adsorption of extract constituents onto electrochemically active sites on the steel surface. By occupying these sites, the adsorbed species reduce the effective surface area available for iron dissolution and consequently decrease the anodic current density. Concurrently, the adsorbed layer can cover cathodically active sites and impede the interfacial electron-transfer processes associated with the cathodic reactions. The presence of heteroatoms, particularly O and N, as well as π-electron systems and other electron-rich functional groups, may facilitate interactions between the organic constituents of the extract and the steel surface. These interactions favor the development of an adsorbed interfacial film that acts as a barrier to the transport of aggressive species toward the metal surface. In particular, the film may hinder the access of H^+^ ions to cathodically active sites, thereby suppressing the kinetics of hydrogen evolution. Under naturally aerated conditions, the same protective layer may also hinder the transport of dissolved O_2_ toward the steel surface, contributing to the inhibition of oxygen reduction. Overall, the simultaneous suppression of the anodic and cathodic partial reactions accounts for the substantial decrease in I_corr_ and demonstrates the effective corrosion-protection capability of *Neolentinus lepideus* extract for AISI 1018 carbon steel in 0.5 M H_2_SO_4_.

The lowest I_corr_ value was obtained at an extract concentration of 400 ppm of *Neolentinus lepideus*. For comparison, Haldhar et al. [17] reported an I_corr_ value of 5.3 × 10^−7^ mA cm^−2^ using 500 ppm of *Valeriana wallichii* root extract as a green corrosion inhibitor for mild steel in 0.5 M H_2_SO_4_. Similarly, Zhou et al. [18] reported an I_corr_ value of 7.2 × 10^−8^ mA cm^−2^ when 400 ppm of waste feverfew root extract was employed. In contrast, Elabbasy et al. [27] obtained a substantially higher I_corr_ value of 0.062 mA cm^−2^ using 300 ppm of *Rosa damascena* extract under comparable acidic conditions. The I_corr_ obtained in the present study therefore falls within the range of values reported for green corrosion inhibitors of carbon and mild steel in sulfuric acid media, further supporting the effectiveness of *Neolentinus lepideus* extract in mitigating steel corrosion.

#### 2.4.3. Electrochemical Impedance Spectroscopy Results

**Figure 8:**
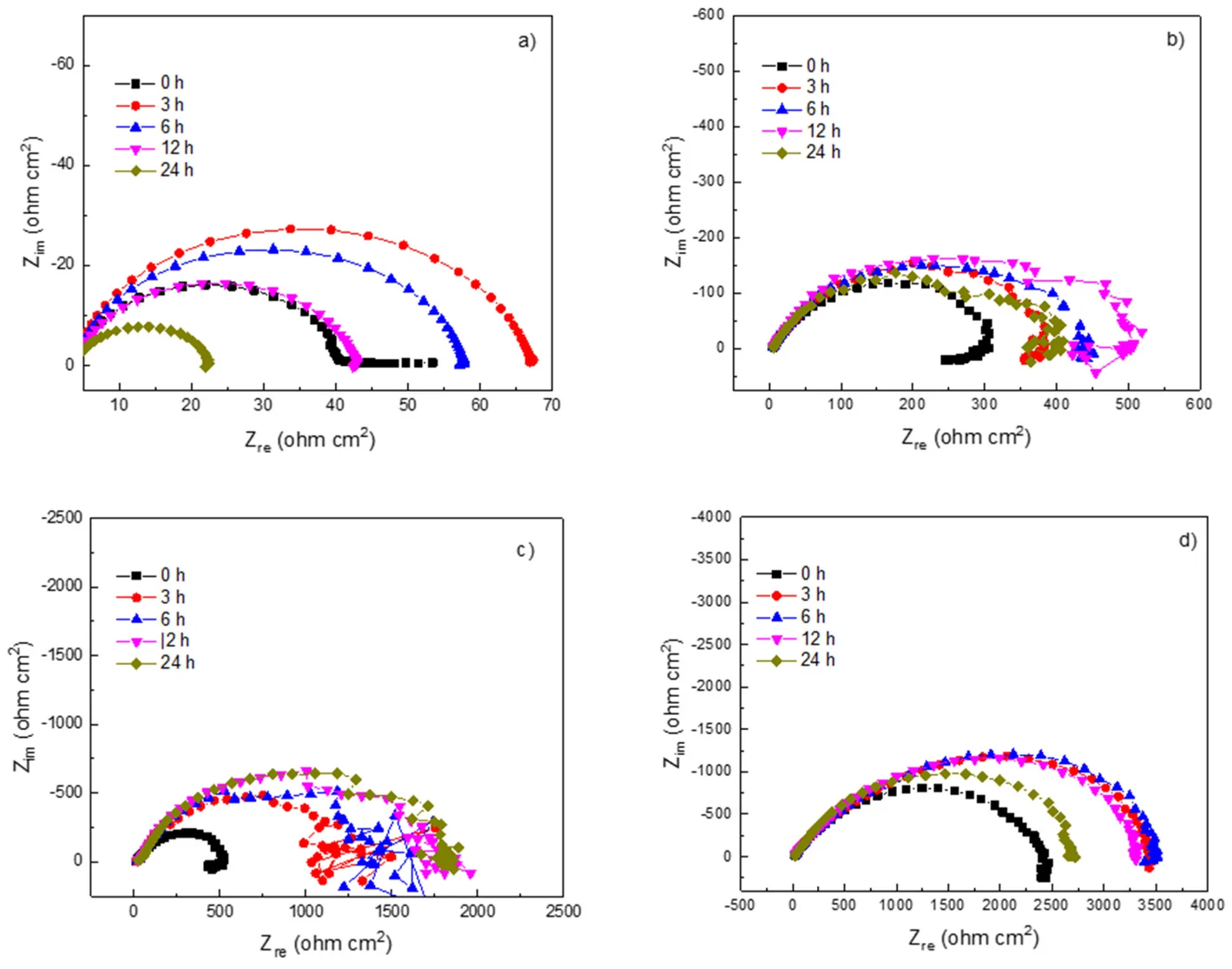
Nyquist plots for 1018 carbon steel in 0.5 M H_2_SO_4_ with the addition of (**a**) 0, (**b**) 100, (**c**) 200 and (**d**) 400 ppm of *Neolentinus lepideus* extract.

The effect of *Neolentinus lepideus* extract concentration on the Nyquist diagrams as a function of time is shown in Figure 8.

For the specimen exposed to the uninhibited solution (Figure 8a), the Nyquist plot exhibits a single depressed capacitive semicircle over the entire frequency range, indicating that the corrosion process is predominantly controlled by charge transfer. The diameter of the capacitive semicircle initially increases with exposure time, reaching a maximum after 3 h. However, a subsequent decrease is observed at longer exposure times, which may be associated with the partial desorption, dissolution, or degradation of the corrosion-product layer formed on the steel surface.

Upon addition of 100 and 200 ppm of *Neolentinus lepideus* extract (Figure 8b,c), a distinct modification in the impedance response is observed. The Nyquist plots display a depressed capacitive semicircle at high and intermediate frequencies, followed by an inductive loop in the low-frequency region. The appearance of the inductive feature suggests that the corrosion mechanism is influenced by the adsorption and desorption of intermediate species at the metal/solution interface [37,38,39]. In addition, the diameter of the low-frequency capacitive response increases markedly with increasing inhibitor concentration. Whereas the uninhibited system exhibits a diameter of approximately 70 ohm cm^2^, values of about 500 and 2000 ohm cm^2^ are obtained in the presence of 100 and 200 ppm of *Neolentinus lepideus* extract, respectively. This substantial increase in impedance indicates enhanced resistance to charge transfer and, consequently, the formation of a more protective interfacial layer in the presence of the extract. Moreover, at these inhibitor concentrations, the diameter of the capacitive response progressively increases with exposure time, suggesting a gradual increase in the thickness and/or compactness of the protective film formed on the steel surface.

At the highest inhibitor concentration investigated, 400 ppm (Figure 8d), the Nyquist plot exhibits a single depressed capacitive semicircle extending over the entire frequency range. This response indicates that the corrosion process is predominantly governed by charge-transfer reactions, with no discernible inductive contribution associated with the adsorption/desorption of intermediate species, as observed at the lower inhibitor concentrations. Notably, the semicircle diameter is approximately 2500 ohm cm^2^ at the initial stage of exposure, which is substantially higher than that recorded for the uninhibited system and those containing lower concentrations of the extract. Furthermore, the diameter progressively increases with exposure time, indicating a continuous enhancement of the interfacial resistance and the development of an increasingly stable and protective film on the steel surface. These results demonstrate that 400 ppm of *Neolentinus lepideus* extract promotes the formation and persistence of a highly resistive protective layer, thereby effectively suppressing the electrochemical corrosion reactions.

**Figure 9:**
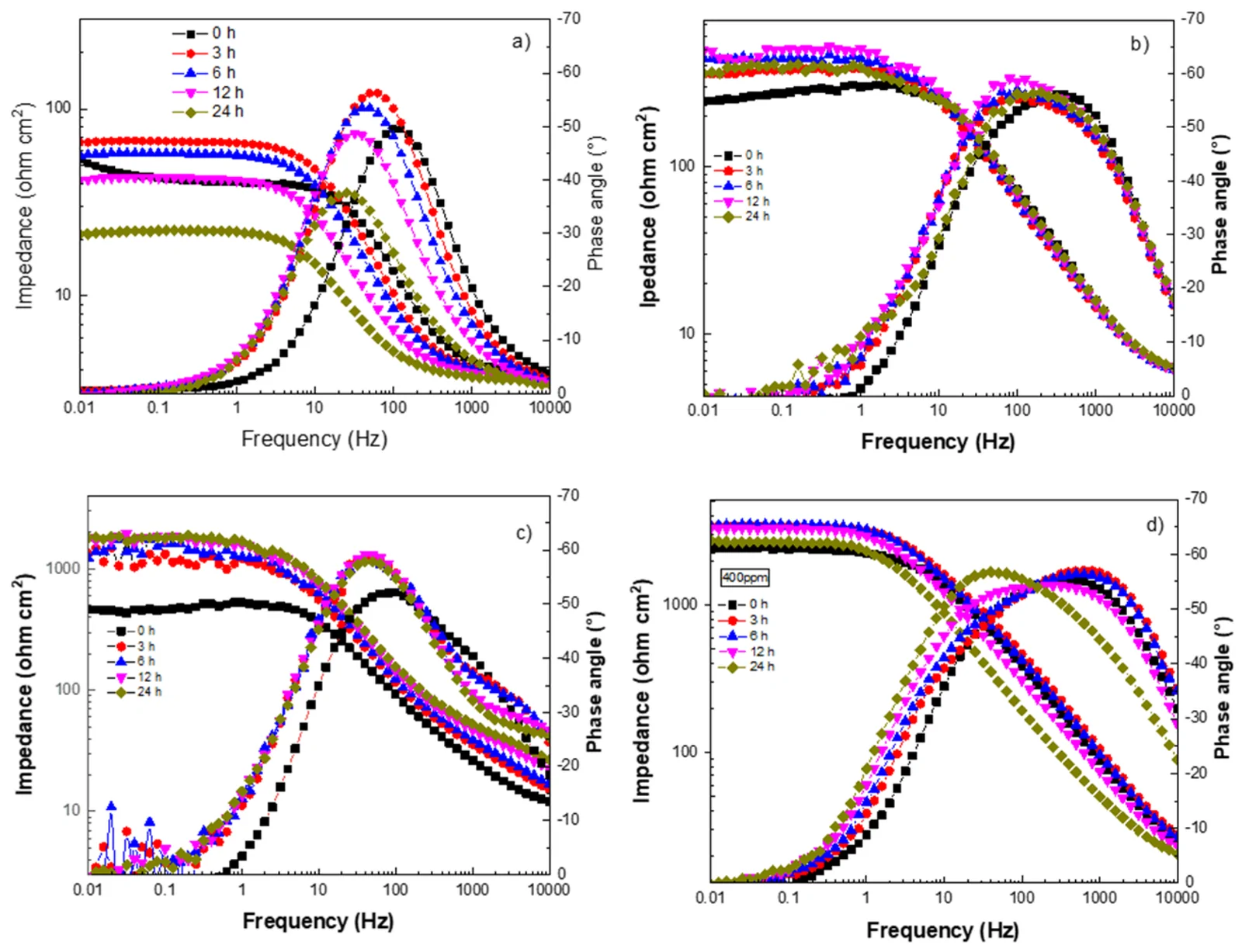
Bode plots for 1018 carbon steel in 0.5 M H_2_SO_4_ with the addition of (**a**) 0, (**b**) 100, (**c**) 200 and (**d**) 400 ppm of *Neoentinus lepideus* extract.

On the other hand, the Bode plots obtained for the uninhibited solution (Figure 9a) show that the impedance magnitude initially increases during the first hours of exposure, followed by a gradual decrease with increasing exposure time. The phase-angle plot exhibits a single maximum, corresponding to one dominant time constant, which is consistent with an electrochemical response primarily associated with charge-transfer processes at the metal/solution interface. The subsequent decrease in impedance suggests a deterioration of the interfacial layer formed during corrosion and, consequently, a progressive loss of surface protection.

In the presence of *Neolentinus lepideus* extract (Figure 9b–d), the impedance magnitude is considerably higher than that recorded for the uninhibited system and generally increases with both inhibitor concentration and exposure time. This behavior indicates an increase in the resistance of the metal/solution interface to electrochemical reactions. Furthermore, the phase-angle plots exhibit two distinct maxima, which become broader and more pronounced as the extract concentration increases. The appearance of two time constants can be attributed to the contribution of two electrochemical processes occurring at different characteristic relaxation frequencies, namely, charge transfer at the metal/solution interface and the response of the protective surface layer. These results support the formation of a more stable and protective interfacial film in the presence of *Neolentinus lepideus* extract.

**Figure 10:**
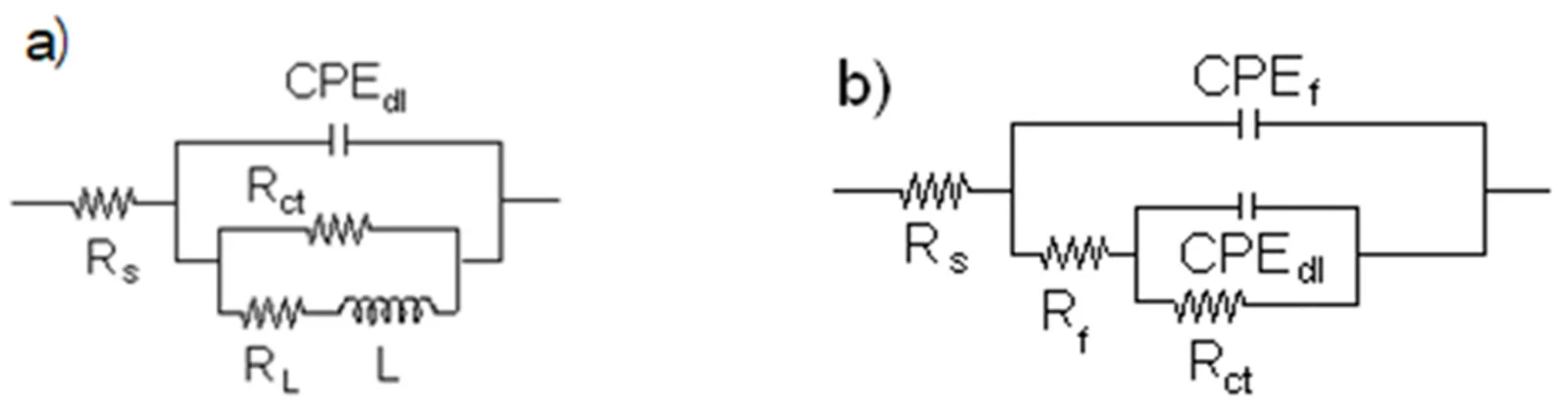
Equivalent electric circuits used to simulate EIS data for carbon steel corroded in 0.5 M H_2_SO_4_ containing (**a**) 0 or 400, and (**b**) 100 or 200 ppm of *Neolentinus lepideus* extract.

The metal/solution interface can be considered an electrified interfacial region in which several electrochemical processes occur simultaneously. These processes can be represented by an appropriate equivalent electrical circuit consisting of resistive, capacitive, and inductive elements, as illustrated in Figure 10. In this circuit, R_s_ represents the solution resistance, whereas R_ct_ corresponds to the charge-transfer resistance associated with the electrochemical reactions occurring at the metal/solution interface. The double-layer capacitance is represented by C_dl_. In systems where a protective corrosion-product/inhibitor film is formed, R_f_ represents the resistance of this surface film, while C_f_ accounts for its associated capacitance. The inductive element, L, describes the inductive response associated with the adsorption/desorption of intermediate species, and R_L_ represents the corresponding resistance.

In practice, the metal/electrolyte interface does not behave as an ideal capacitor because of surface heterogeneities arising from factors such as surface roughness, no uniform corrosion, variations in the composition and thickness of the surface film, and localized dissolution. Therefore, the ideal capacitive elements are commonly replaced by constant phase elements (CPEs), which provide a more realistic representation of the non-ideal capacitive behavior of the interface. The impedance of a CPE, Z_CPE_, is expressed as:

Z_CPE_ = Y^−1^ (*iw*)^−n^ (9)

where Y is the CPE admittance, *i* is the imaginary unit √−1, *w* is 2πf, f is the frequency, and n is the CPE exponent, which describes the degree of deviation from ideal capacitive behavior [37,38,39].

The electrochemical parameters obtained from fitting the experimental EIS data using the equivalent circuits shown in Figure 10 are summarized in Table 4. The results reveal a progressive increase in R_ct_ with increasing *Neolentinus lepideus* extract concentration. This increase in charge-transfer resistance indicates a decrease in the rate of electrochemical charge-transfer reactions associated with metal dissolution and/or the cathodic reaction. The higher R_ct_ values therefore confirm that adsorption of the extract constituents and the subsequent formation of a protective interfacial film hinder the access of aggressive species to the steel surface and impede the corrosion reactions. Consequently, the presence of *Neolentinus lepideus* extract increases the resistance of the metal/solution interface and enhances the corrosion protection of the steel.

The adsorption of inhibitor molecules modifies the structure and physicochemical properties of the electrical double layer, thereby reducing the number of electrochemically active sites available for corrosion reactions and altering the interfacial environment. This interpretation is consistent with the EIS results, which show an increase in R_ct_ accompanied by a decrease in the double-layer CPE admittance, Y_dl_. A decrease in the capacitive response of the interface is commonly associated with an increase in the effective thickness of the interfacial layer and/or a decrease in its effective dielectric constant. For an idealized electrical double layer, its capacitance can be expressed as:

C_dl_ = (εε_0_/λ)A (10)

where ε_0_ is the vacuum permittivity, ε is the effective dielectric constant of the interfacial medium, λ represents the effective thickness of the electrical double layer, and A is the exposed electrode area [40]. According to Equation (10), a decrease in C_dl_ may therefore result from an increase in λ and/or a decrease in the effective dielectric constant of the interfacial region.

Upon addition of *Neolentinus lepideus* extract to the electrolyte, its organic constituents can adsorb onto the steel surface and partially displace pre-adsorbed water molecules from the interface into the bulk solution. The replacement of water molecules by adsorbed organic species modifies the dielectric properties and effective thickness of the interfacial region. Because the adsorbed inhibitor molecules form an organic layer at the metal/electrolyte interface, their presence can increase the effective separation between the metallic surface and the ionic species in the electrolyte, thereby contributing to the observed decrease in the capacitive response. Thus, the simultaneous increase in R_ct_ and decrease in Y_dl_ are consistent with the formation of an adsorbed protective layer that inhibits charge-transfer reactions at the steel/electrolyte interface.

At 400 ppm, the progressive increase in R_ct_ with exposure time indicates that the inhibiting effect of *Neolentinus lepideus* extract becomes more pronounced during immersion. This behavior suggests a time-dependent modification of the steel/electrolyte interface, most likely associated with the progressive accumulation and/or adsorption of extract constituents at the metal surface. As the interfacial layer becomes more continuous, the accessibility of the corrosive electrolyte to the steel surface is expected to decrease, resulting in greater resistance to the electrochemical charge-transfer reactions and, consequently, higher R_ct_ values.

The evolution of the film-related resistance R_f_ provides additional information on this interfacial process. An increase in R_f_ with exposure time would indicate a progressive increase in the resistance of the surface layer to ionic transport, consistent with the development of a more effective barrier at the steel/electrolyte interface. Conversely, changes in Y_dl_ can be associated with modifications of the interfacial region resulting from inhibitor adsorption. A decrease in Y_dl_ with exposure time would be consistent with a reduction in the effective electrochemically active area and/or changes in the dielectric properties of the interface as the surface becomes increasingly covered by extract constituents. Therefore, the simultaneous evolution of R_ct_, R_f_, and Y_dl_ with immersion time supports a progressive enhancement of the interfacial barrier effect at 400 ppm.

In addition, the n_dl_ parameter provides valuable information regarding the degree of non-ideal capacitive behavior of the metal/electrolyte interface and can be related to changes in surface heterogeneity. Lower n_dl_ values generally indicate greater deviation from ideal capacitive behavior, which may arise from surface roughness, no uniform corrosion, defects, and other interfacial heterogeneities, whereas values approaching unity are indicative of a more homogeneous interfacial response. As shown in Table 3, the uninhibited system exhibits an n_dl_ value of approximately 0.7, indicating a relatively heterogeneous interface, which is consistent with the significant surface degradation associated with the uninhibited corrosion process. In contrast, the n_dl_ value increases to approximately 0.9 at 400 ppm of *Neolentinus lepideus* extract, indicating a substantially more homogeneous interfacial response. This increase suggests that adsorption of the extract constituents suppresses localized metal dissolution and contributes to the development of a more uniform protective layer over the steel surface.

Overall, the EIS parameters demonstrate that the protective effect of *Neolentinus lepideus* extract becomes more pronounced with increasing inhibitor concentration and exposure time. The progressive increase in R_ct_ and R_f_, together with the decrease in Y_dl_ and the increase in n_dl_, indicates that adsorption of the extract constituents modifies the metal/electrolyte interface and promotes the development of a more resistive and homogeneous protective layer. The continuous increase in film resistance with exposure time further suggests that the interfacial layer undergoes progressive development, resulting in greater surface coverage and enhanced resistance to the electrochemical reactions associated with steel corrosion.

### 2.5. Surface Analysis

**Figure 11:**
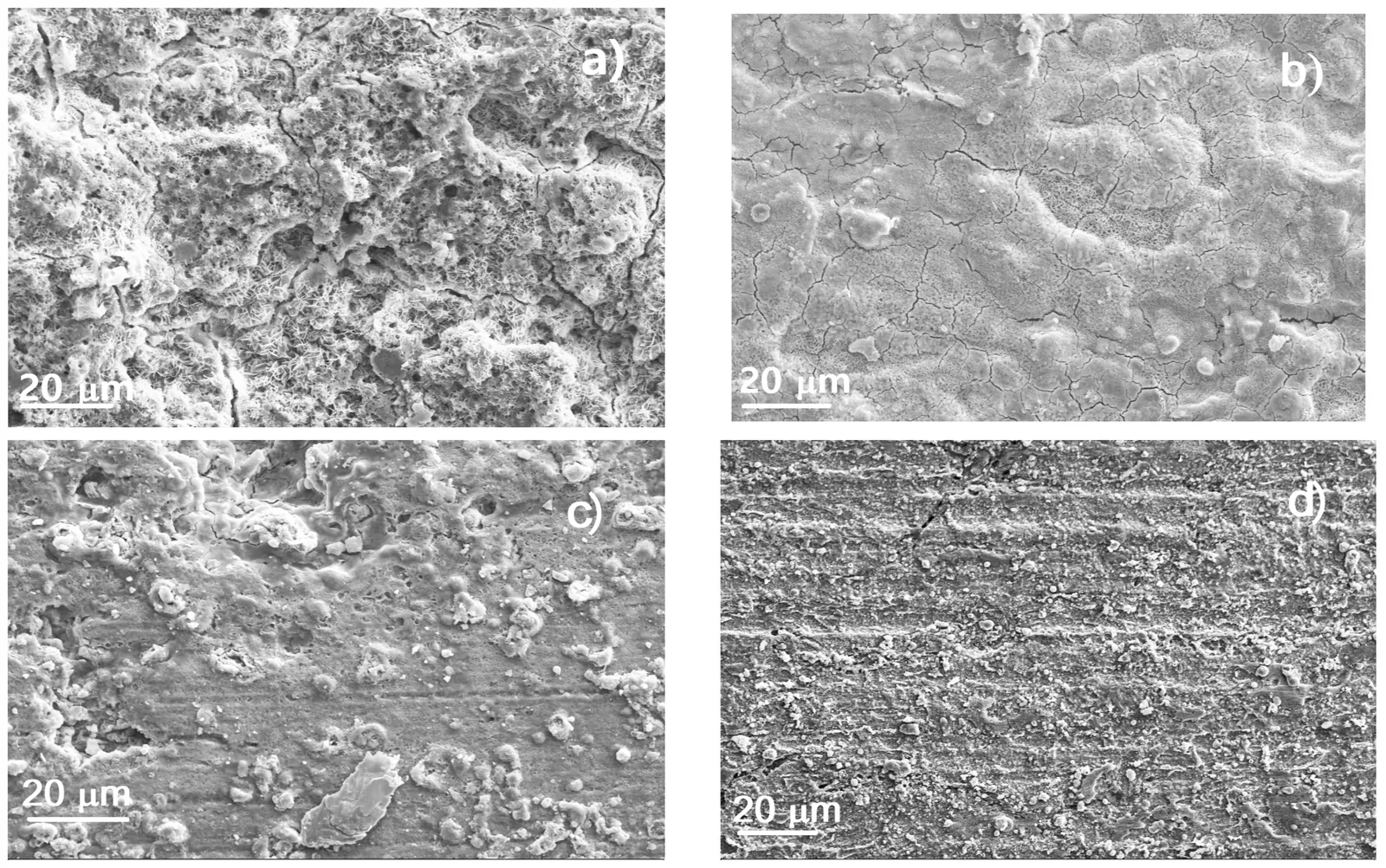
Micrographs of carbon steel corroded in 0.5 M H_2_SO_4_ containing (**a**) 0, (**b**) 100, (**c**) 200 and (**d**) 400 ppm of *Neolentinus lepideus* extract.

The corroded specimens were examined by scanning electron microscopy (SEM) to evaluate the effect of *Neolentinus lepideus* extract on the surface morphology of AISI 1018 carbon steel after exposure to the corrosive medium. The corresponding SEM micrographs are presented in Figure 11a–d. The specimen exposed to the uninhibited solution (blank) (Figure 11a) exhibits a heterogeneous and highly defective surface, characterized by numerous micro cracks, pores, and irregular corrosion features. Such morphological defects may facilitate the access and transport of aggressive electrolyte species toward the underlying metal surface, favoring continued corrosion.

Upon addition of *Neolentinus lepideus* extract (Figure 11b,c), a progressive decrease in the density and extent of surface cracks and pores is observed, together with the development of a comparatively more compact and homogeneous surface morphology. This change indicates a lower degree of surface degradation in the presence of the extract and is consistent with the inhibition behavior observed from the gravimetric and electrochemical measurements. At the highest inhibitor concentration investigated (400 ppm) (Figure 11d), the surface appears considerably more compact and uniform, with substantially fewer visible cracks, pores, and other corrosion-related defects than those observed for the uninhibited specimen and at lower extract concentrations.

Although SEM examination alone does not allow the chemical composition or specific nature of the surface layer to be established, the observed morphological changes are consistent with the formation or retention of a more continuous surface layer in the presence of *Neolentinus lepideus* extract. The reduced number of surface defects at increasing extract concentration may decrease the accessibility of the corrosive electrolyte to the steel substrate, thereby contributing to the enhanced corrosion inhibition observed at higher inhibitor concentrations. These observations provide morphological evidence supporting the protective effect of *Neolentinus lepideus* extract, while further surface-chemical characterization would be required to determine the composition and specific chemical interactions associated with the surface layer.

### 2.6. Corrosion Inhibition Mechanism

**Figure 12:**
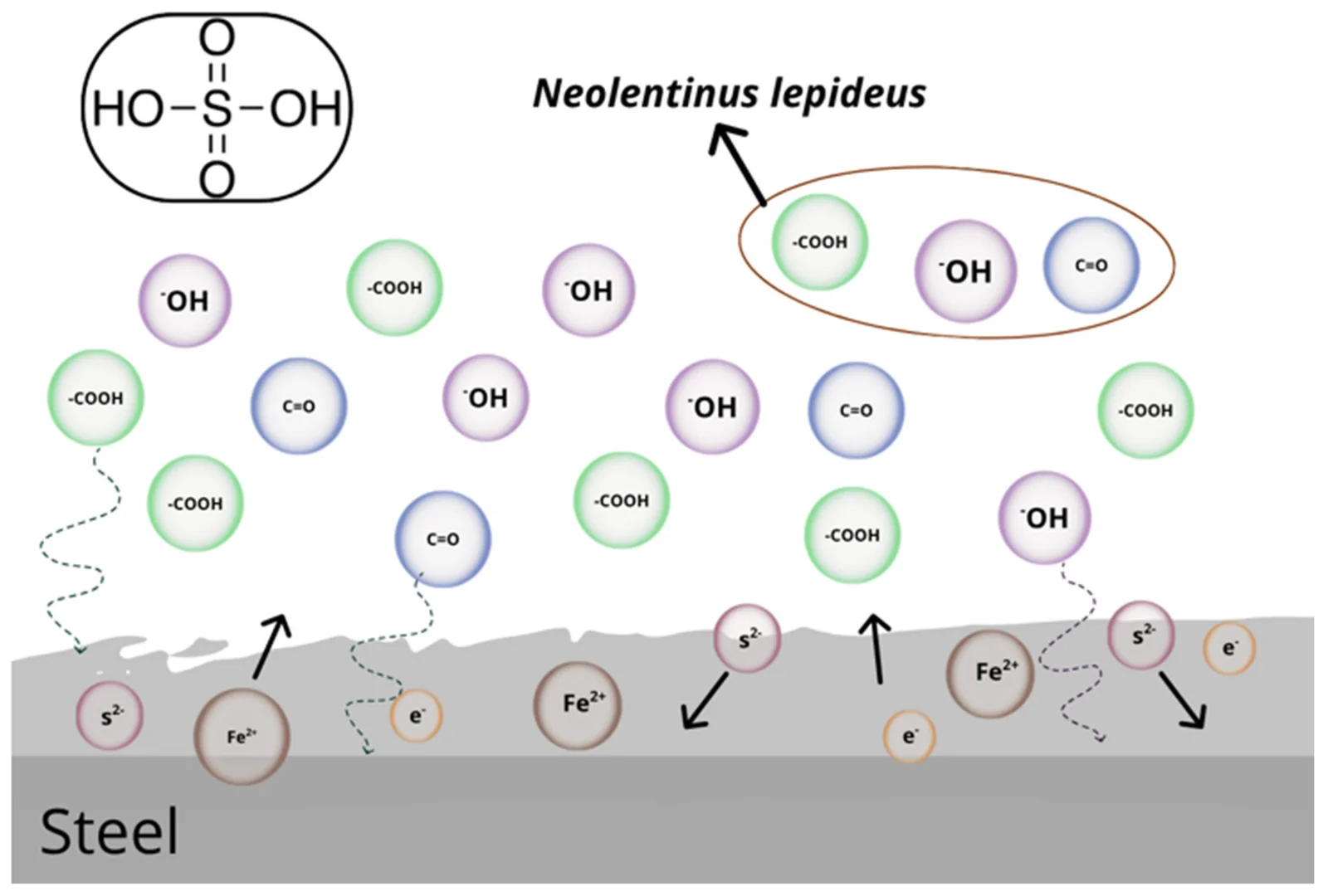
Corrosion inhibition mechanism for carbon steel in 0.5 M H_2_SO_4_ by *Neolentinus lepideus* extract.

Gravimetric and electrochemical results indicate that *Neolentinus lepideus* extract acts as a mixed-type corrosion inhibitor for AISI 1018 carbon steel in 0.5 M H_2_SO_4_ by simultaneously retarding the anodic dissolution of iron and the cathodic hydrogen evolution reaction. The inhibition effect can be attributed primarily to the adsorption of organic constituents present in the mushroom extract onto the steel surface, resulting in the formation of a protective adsorbed film. This film reduces the exposure of the metallic surface to the acidic electrolyte and consequently limits the electrochemical reactions responsible for corrosion. In the absence of the inhibitor, corrosion proceeds through the anodic dissolution of iron according to Fe → Fe^2+^ + 2e^−^, accompanied by the cathodic reduction of hydrogen ions and/or water to produce molecular hydrogen. Upon addition of the *Neolentinus lepideus* extract, its organic constituents interact with the steel/electrolyte interface through heteroatoms and π-electron systems, particularly those associated with functional groups identified by FTIR analysis, such as hydroxyl, carbonyl, and amide groups. These electron-donating centers can interact with the metal surface as illustrated in Figure 12, and promote the formation of an adsorbed protective layer. The adsorption of the organic constituents can occur through electrostatic interactions and, depending on the chemical nature of the adsorbed species and the steel surface, through donor–acceptor interactions between electron-rich functional groups and vacant or partially vacant iron surface sites. In addition, intermolecular interactions among the adsorbed organic molecules may contribute to the stabilization and compactness of the protective layer. The resulting adsorbed film acts as a physical barrier that decreases the number of active sites available for both iron dissolution and hydrogen evolution. This interpretation is consistent with the increase in polarization resistance and the decrease in corrosion current density observed with increasing extract concentration. At the optimum concentration, greater surface coverage by the extract constituents is expected, leading to a more continuous and compact protective layer and, consequently, enhanced corrosion inhibition. The decrease in inhibition efficiency at elevated temperature may further indicate a weakening of the adsorbed layer and/or partial desorption of physically adsorbed species from the steel surface. Overall, the corrosion inhibition mechanism of *Neolentinus lepideus* extract can therefore be described as predominantly adsorption-controlled, involving the formation of an organic protective film that blocks active sites and suppresses both anodic iron dissolution and cathodic hydrogen evolution.

## 3. Materials and Methods

### 3.1. Testing Material

The material investigated in this study was AISI 1018 carbon steel supplied in the form of cylindrical bars with a diameter of 6.00 mm. The chemical composition of the steel was as follows (wt.%): 0.18 C, 0.75 Mn, 0.04 P, 0.05 S, with Fe as the balance. Cylindrical specimens with a length of 10.0 mm were sectioned from the bars and used for both gravimetric (weight-loss) and electrochemical measurements.

### 3.2. Electrolyte Preparation

Fresh specimens of *Neolentinus lepideus* were collected from a forested area, thoroughly washed with distilled water, and dried at room temperature. Subsequently, 1000 g of the fungal material were macerated in 1000 mL of methanol for 30 days. The methanolic extract was filtered through Whatman No. 1 filter paper to remove insoluble residues. The resulting filtrate was concentrated by rotary evaporation at 50 °C under reduced pressure (100 mbar) at a rotation speed of 100 rpm. The concentrated extract was subsequently dried in a desiccator over silica gel until a constant mass was achieved. The resulting dried crude extract was weighed and stored in an amber glass container at 4 °C until further use. The extraction yield would be determined gravimetrically by dividing the mass of the dried extract by the initial mass of the starting material and multiplying by 100.

Based on these considerations, the use of Soxhlet extraction with a 50:50 (*v*/*v*) water–ethanol mixture could represent a more suitable alternative, as it would reduce solvent toxicity while substantially shortening the extraction time. Following the maceration period, the mixture was processed to obtain a solid extract. A 0.1 g portion of the resulting extract was dissolved in 100 mL of methanol to prepare the stock solution. Appropriate aliquots of the stock solution were then diluted to obtain final inhibitor concentrations of 100, 200, and 400 ppm. A solution without inhibitor (0 ppm) was used as the blank. The prepared inhibitor solutions were added to 0.5 M H_2_SO_4_, which was prepared using analytical-grade reagents and distilled water. The *Neolentinus lepideus* extract was characterized by Fourier-transform infrared (FTIR) spectroscopy using a Bruker Tensor 27 FTIR spectrometer to identify the principal functional groups and chemical constituents present in the extract.

### 3.3. Electrochemical Techniques

Both potentiodynamic polarization (PDP) and electrochemical impedance spectroscopy (EIS) measurements were employed to investigate the corrosion behavior of AISI 1018 carbon steel in the absence and presence of *Neolentinus lepideus* extract. Prior to each electrochemical measurement, the open-circuit potential (OCP) was monitored for 30 min to allow the metal/electrolyte interface to attain a stable state. All electrochemical measurements were performed in a conventional three-electrode glass cell open to the atmosphere, using the steel specimen as the working electrode, a silver/silver chloride (Ag/AgCl) electrode as the reference electrode, and a graphite rod as the counter electrode. Prior to testing, the steel specimens were embedded in polymeric resin and mechanically abraded using 600-grit SiC abrasive paper. The specimens were subsequently rinsed thoroughly with distilled water and acetone and dried under a gentle stream of warm air. Potentiodynamic polarization measurements were initiated at a potential 500 mV more negative than the corrosion potential E_corr_. The potential was then swept in the anodic direction at a scan rate of 0.5 mV s^−1^ until reaching a potential 700 mV more positive than E_corr_. The corrosion current density I_corr_ was manually determined from the polarization curves using the Tafel extrapolation method using a region of ±250 mV around the E_corr_ value. Electrochemical impedance spectroscopy measurements were conducted at the stabilized E_corr_ by applying a sinusoidal perturbation of ±15 mV. The impedance response was recorded over a frequency range from 10,000 to 0.01 Hz. The resulting spectra were analyzed to characterize the interfacial electrochemical processes and assess the effect of *Neolentinus lepideus* extract on the corrosion behavior of AISI 1018 carbon steel. All tests were repeated 3 times.

### 3.4. Weight Loss Tests

For the weight-loss measurements, cylindrical 1018 carbon steel specimens measuring 10.0 mm in length and 6.0 mm in diameter were prepared. The specimens were mechanically ground using 1200-grit SiC abrasive paper, thoroughly rinsed with distilled water and acetone, and subsequently dried with warm air. For each inhibitor concentration, three specimens were immersed in separate glass flasks containing 0, 100, 200, or 400 ppm of *Neolentinus lepideus* extract. The experiments were conducted at 25, 40, and 60 °C for 72 h. After each immersion period, the specimens were removed, rinsed, cleaned to eliminate corrosion products, dried, and weighed. The weight loss (ΔM) was determined from the difference between the initial and final masses of each specimen, as follows:

ΔM = M_i_ − M_f_ (11)

where M_i_ and M_f_ denote the initial and final masses of the specimens before and after immersion in the electrolyte, respectively. The corrosion rate (*CR*), expressed in g cm^2^ h^−1^, was calculated for each inhibitor concentration using the following equation:

*CR* = (ΔM_b_ − ΔM_inh_)/At (12)

where ΔM_b_ represents the weight loss measured in the blank (uninhibited) solution, while ΔM_inh_ corresponds to the weight loss measured in the presence of the inhibitor. (A) denotes the exposed specimen area (cm^2^), and (t) is the immersion time (h). Although this expression represents the difference between two mass losses rather than the corrosion rate of an individual specimen, it has widely used to calculate the corrosion rate [18,19,21,27]. After the immersion tests, the corroded specimens were examined using a LEO scanning electron microscope (SEM) operated under low-vacuum conditions to characterize the morphology of the steel surfaces.

## 4. Conclusions

The *Neolentinus lepideus* extract exhibited pronounced corrosion inhibition performance toward AISI 1018 carbon steel in 0.5 M H_2_SO_4_. The inhibition efficiency increased with increasing extract concentration, reaching a maximum value of 89% at 400 ppm, whereas a progressive decrease in inhibition efficiency was observed with increasing temperature. The extract exhibited mixed-type inhibition behavior, with a predominant influence on the cathodic reactions, particularly oxygen reduction and hydrogen evolution. The calculated adsorption free-energy values indicated that the interaction of *Neolentinus lepideus* extract with the steel surface occurs predominantly through physisorption and is consistent with the Freundlich adsorption isotherm. Electrochemical impedance measurements indicated that the corrosion process was predominantly controlled by charge transfer. The addition of 400 ppm extract did not substantially modify the prevailing corrosion mechanism, whereas at lower concentrations (100 and 200 ppm), the electrochemical response suggested the involvement of adsorption/desorption processes of intermediate species at the steel/solution interface. The addition of *Neolentinus lepideus* extract resulted in an increase in charge-transfer resistance and a concomitant decrease in double-layer capacitance, supporting the formation of an adsorbed protective layer on the steel surface. The inhibitory effect of the extract can be associated with its content of phenolic compounds, amides, and polysaccharides, whose functional groups can interact with the metal surface and contribute to the formation of a protective film, thereby hindering the electrochemical reactions involved in the corrosion process. The strongest commercial argument is that the extract combines high inhibition efficiency with a renewable biological source, and this makes it a good potential corrosion inhibitor. However, the principal barriers to commercialization are batch-to-batch variability, extract standardization, extraction cost, long-term stability, regulatory requirements, and scale-up.

## Figures and Tables

**Table 1:** Comparison between inhibitor efficiency values obtained by *Neolentinus lepideus* extract and other green corrosion inhibitors for carbon steel in 0.5 M H_2_SO_4_.

Reference	Inhibitor Extract	Concentration (ppm)	Inhibitor Efficiency (%)
Haldhar et al. [17]	*Valeriana wallichii* root	500	93.4
Zhou et al. [18]	waste feverfew root	400	96
Ikeuba et al. [19]	Expired aspirin	800	79
Wang et al. [28]	Pectin-derived carbon dots and 1-hexylamine, 1,2-diaminocyclohexane, and diethylenetriamine	200	75.3, 90.1, and 95.4
Elabbasy et al. [27]	*Rosa damascena*	300	87
This work	*Neolentinus lepideus*	400	89

**Table 2:** Thermodynamic parameters for 1018 carbon steel exposed to 0.5 M H_2_SO_4_ with the addition of *Neolentinus lepideus* extract.

C_inh_ (ppm)	*E_a_* (kJ mol^−1^)	ΔH* (kJ mol^−1^)	ΔS* (J mol^−1^K^−1^)
0	10.3	3.53	−259.91
50	13.7	3.99	−260.25
100	20.4	8.74	−246.49
200	30.3	16.89	−222.71
400	38.8	27.77	−192.42

**Table 3:** Electrochemical parameters obtained from polarization curves.

C_inh_ (ppm)	E_corr_ (mV)	I_corr_ (mA/cm^2^)	R_p_ (ohm cm^2^)	β_a_ (mV/dec)	β_c_ (mV/dec)	I.E. (%)	θ
0	−490	1.0 × 10^−3^	53	160	560	---	---
100	−530	1.4 × 10^−5^	307	120	250	99	0.99
200	−530	1.5 × 10^−6^	510	115	220	99	0.99
400	−530	9.0 × 10^−7^	2495	110	210	99	0.99

Standard deviation in E_corr_ is ± 5 mV, in I_corr_ ± 1.1 × 10^−7^ and in R_p_ ± 5 ohm cm^2^.

**Table 4:** Electrochemical parameters used to fit EIS data for 1018 carbon steel exposed to 0.5 M H_2_SO_4_ with the addition of *Neolentinus lepideus* extract.

Time (h)	C_inh_ (ppm)	Y_dl_ (F cm^−2^)	n_dl_	R_ct_ (ohm cm^2^)	Y_f_ (F cm^−2^)	n_f_	R_f_ (ohm cm^2^)	L (H·cm^2^)	R_L_ (ohm cm^2^)	I.E. (%)
0	0	6.25 × 10^−4^	0.7	34	-----	0.7	-----	-----	-----	-----
	100	5.94 × 10^−4^	0.8	277	3.35 × 10^−4^	0.8	32	12	11	87.7
	200	1.76 × 10^−4^	0.9	345	2.25 × 10^−4^	0.8	95	53	196	90.1
	400	1.43 × 10^−5^	0.9	1397	8.88 × 10^−6^	0.8	171	----	-----	97.7
3	0	5.05 × 10^−4^	0.7	63	-----	-----	-----	-----	-----	-----
	100	4.07 × 10^−4^	0.8	361	3.15 × 10^−4^	0.8	43	26	15	82.5
	200	2.16 × 10^−4^	0.8	998	2.06 × 10^−5^	0.9	105	62	58	93.7
	400	1.22 × 10^−5^	0.9	1803	1.24 × 10^−5^	0.9	315	------	------	97.3
6	0	5.53 × 10^−4^	0.7	54	-----	-----	-----	-----	-----	-----
	100	4.01 × 10^−4^	0.8	430	3.05 × 10^−4^	0.8	56	6	18	87.4
	200	2.64 × 10^−4^	0.8	1134	2.70 × 10^−5^	0.8	175	55	47	95.2
	400	1.13 × 10^−5^	0.9	2220	1.29 × 10^−5^	0.9	741	-----	-----	97.6
12	0	7.73 × 10^−4^	0.7	39	-----	-----	-----	-----	-----	-----
	100	3.94 × 10^−4^	0.8	455	9.25 × 10^−5^	0.9	60	68	11	91.4
	200	3.79 × 10^−5^	0.8	1608	1.54 × 10^−5^	0.9	255	76	55	97.6
	400	1.04 × 10^−5^	0.9	2539	1.27 × 10^−5^	0.8	1022	-----	-----	98.4
24	0	8.62 × 10^−4^	0.7	19	-----	-----	-----	-----	-----	-----
	100	3.02 × 10^−4^	0.8	365	2.06 × 10^−5^	0.9	98	92	24	94.7
	200	2.84 × 10^−5^	0.8	1760	1.94 × 10^−5^	0.9	315	201	60	98.9
	400	9.30 × 10^−6^	0.9	2685	1.80 × 10^−5^	0.8	1531	----	----	99.2

## Data Availability

Data that support this research work are available upon request.
